# Monteggia fractures and Monteggia-like-lesions: a systematic review

**DOI:** 10.1007/s00402-022-04576-1

**Published:** 2022-09-03

**Authors:** Marc Maximilian Weber, Thomas Rosteius, Thomas A. Schildhauer, Matthias Königshausen, Valentin Rausch

**Affiliations:** 1 Department of General and Trauma Surgery, BG University Hospital Bergmannsheil, BŸrkle-de-la-Camp-Platz 1, 44789 Bochum, Germany; 2 Faculty of Medicine and University Hospital, Center for Orthopedic and Trauma Surgery, University of Cologne, Kerpener Str. 62, 50937 Cologne, Germany

**Keywords:** Monteggia injury, Elbow trauma, Olecranon fracture, Suture, Radial head fracture, Coronoid fracture, Elbow dislocation, Ulna non-union

## Abstract

Monteggia injuries are rare, but severe injuries of the elbow including various injury patterns. Treatment of these injuries is still topic of debate and strategies differ widely. In this systematic review on Monteggia injuries in adults, we aimed to clarify the incidence of different injury patterns within Monteggia injuries, investigate the main reasons leading to revision surgery and explore which surgical treatments should be favored to achieve satisfactory clinical results.

We initially identified 182 publications and ultimately included 17 retrospective studies comprising 651 cases. All patients were classified using the Bado classification, leading to 30.5% Bado type I fractures, 60.4% type II fractures, 5.1% type III and 3.1% type IV fractures. Mean revision rate was 23%. Ulna non-union (28%) and limited range-of-motion (22%) are the main reasons for revision surgery. Meta-analysis shows a trend toward the use of locking plates for ulna fixation which may lead to less revision surgery and fewer ulna non-unions. Further biomechanical and clinical research is necessary to clarify the role of radial head surgery.

## Introduction

In 1814, Giovanni Battista Monteggia first described a Monteggia fracture as a fracture of the proximal ulna combined with a dislocation of the radial head [1]. Overall, Monteggia injuries are rare and account for only 1–5% of all fractures around the elbow [2]. Today, the eponym of Monteggia fracture, Monteggia-like-lesion or Monteggia-lesion includes multiple patterns of injury of the proximal ulna and the radial head [3]. Bado classified Monteggia fractures by defining four subtypes depending on the direction of radial head dislocation [4]. Jupiter subclassified the Bado type II fracture with regard to the location of the ulna fracture and included radial head fractures [5]. Giannicola identified six injury patterns in Monteggia lesions: (1) ulna fracture, (2) radio-humeral dislocation, (3) ulno-humeral dislocation, (4) proximal radio-ulnar dislocation, (5) radial fracture and (6) distal radio-ulnar joint lesion [3]. Today, treatment of these injuries is still a topic of debate resulting in widely different treatment strategies. Also, definition of the various injury patterns is inconsistent throughout the literature. Therefore, due to the rarity of these injuries, it is difficult to compare and evaluate different treatment strategies for these injuries.

Our goal with this systematic review was to clarify the incidence of different injuries within Monteggia injuries such as ulna fractures, coronoid fractures and radial head fractures. Furthermore, we aimed to investigate the main reasons leading to revision surgery and explore whether certain surgical treatments should be favored to achieve satisfactory clinical results.

## Materials and methods

We applied the “Preffered Reporting Items for Systematic Reviews and Meta-Analyses” (PRISMA) guidelines to guarantee methodical transparency. We included all studies published in English or German language since 1990 that used the Bado/Jupiter classification for Monteggia fractures and Monteggia-like-lesions. The research excluded studies without Bado classification, biomechanical studies, or anatomically studies. We also excluded review articles, case reports and studies which lack basic information such as mean follow-up or mean age of patients.

The MEDLINE database using the PubMed interface was investigated using the search terms “Monteggia fracture “OR” Monteggia-like-lesion “OR” Monteggia injury” NOT child NOT pediatric. The research was completed on 15.12.2021.

**Fig. 1:**
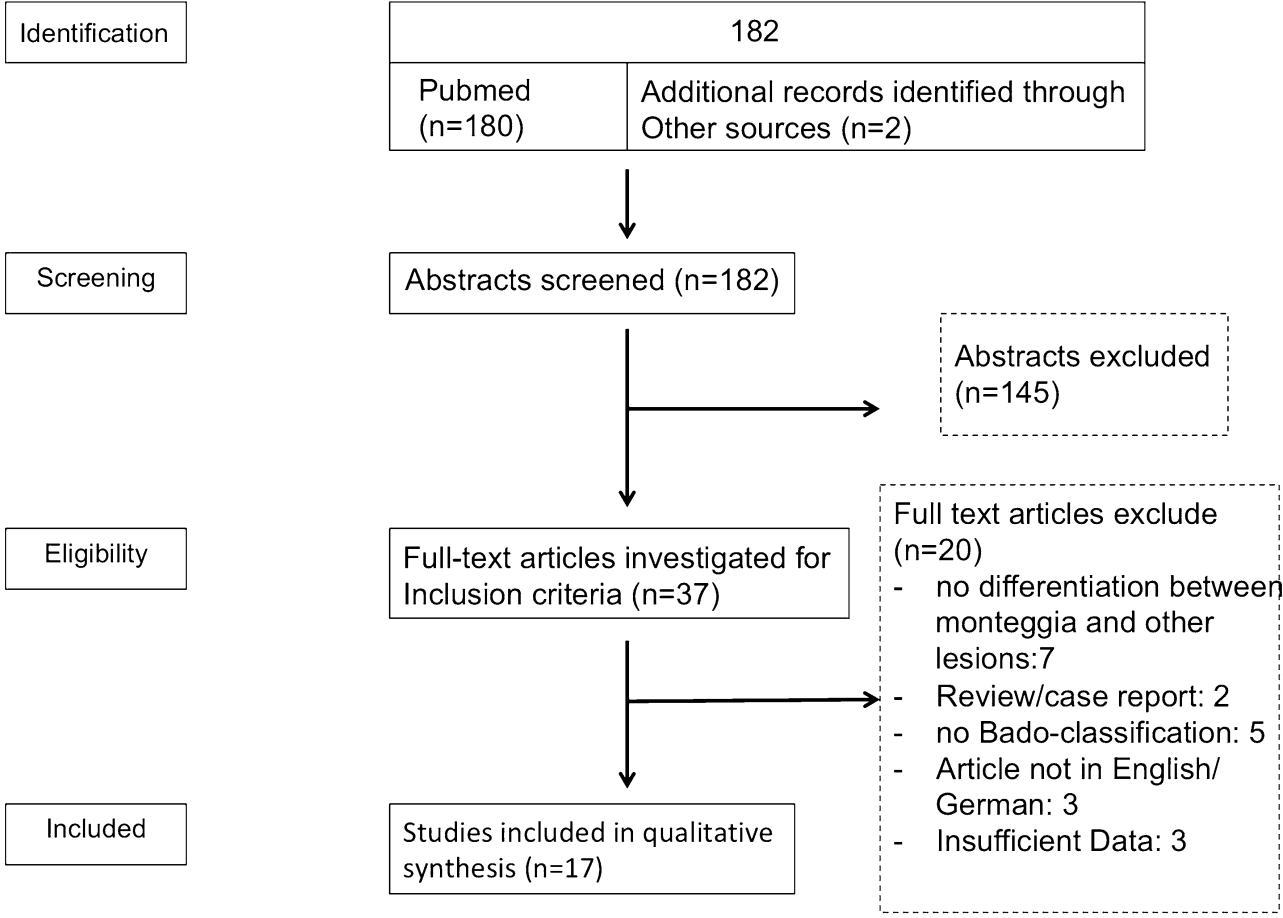
PRISMA-adapted flowchart illustrating study selection and exclusion criteria

The studies identified were examined by two independent reviewers (MMW and VR). First, title and abstracts were checked for exclusion criteria. Subsequently, the full text was evaluated wether inclusion criteria were met by the study. In case of borderline decisions, a third reviewer was consulted to reach a consensus decision (MK). All borderline cases could be resolved by a consensus decision. The process of decision-making is depicted in the PRISMA-adapted flow diagram (Fig. 1).

All data of the 17 included studies regarding number of patients, mean age, gender, mean follow-up, trauma mechanism, fracture classification (Bado/Jupiter, Mason, O’Driscoll, Regan&Morrey), range-of-motion, surgical procedures, outcome scores (*Disability of the arm and shoulder* (DASH); *Mayo Elbow Performance Score* (MEPS); *Mayo Modified Wrist Score* (MMWS); *Broberg and Morrey)*, complications and revision rates were extracted and transferred into tables.

Microsoft^®^ Excel^®^ 2011 for Mac and GraphPad PRISM 6 (Graphpad Software, Inc) were used for data collection, data visualization and statistical analyses.

## Results

We identified 182 studies in relation to our search terms. After exclusion of 145 abstracts that failed to meet the inclusion criteria, 37 full-text articles were assessed for eligibility. As demonstrated in Fig. 1, we excluded 20 articles that did not fulfill the inclusion criteria. Seventeen articles were finally included in qualitative synthesis.

Characteristics of the included publications are depicted in Table 1. In total, 651 patients with a mean age of 48.4 years were included. Gender ratio was nearly balanced with 343 male (53%) and 304 female (47%) reported patients. The mean follow-up was 52.9 months (10–234.5 months). Eleven publications reported the mechanism leading to the Monteggia injury. We identified traffic accidents (35%), low-energy falls (defined as a fall from less than 2 m; 32%) and high-energy falls (14%) as the main reasons for Monteggia injuries. In 52 cases (13%), the height of the fall was not further described. Work-related accidents (4%) and other trauma mechanisms (2%) like gun shots are rare injury causes (Table 2).

**Table 1:** Overview of the publications included, depicting number of patients, mean patient age, gender and mean follow-up in months

First author	Number of patients	Mean age	Male	Female	Mean follow-up (months)
Calderazzi et al. 2020	12	59.25	5	7	17.5
Eden et al. 2019	40	49	23	17	36
Egol et al. 2005	20	52.6	7	13	28.8
Guiton et al. 2009	11	54.6	6	5	234.55
Hamaker et al. 2017	119	37	77	42	10
Jung et al. 2020	27	56	11	16	69
Jungbluth et al. 2017	46	57.7	19	27	65.3
Jupiter et al. 1991	13	56.1	4	9	39.6
Klug et al. 2019	78	54.7	34	44	55.2
Konrad et al. 2007	63	42.1	41	22	100.8
Korner et al. 2004	49	38 (Median)	31	18	83 (Median)
Laun et al. 2015	10	52.4	4	6	12.3
Perez et al. 2002	54	41	37	17	24
Ring et al. 1998	48	52	23	25	78
Schmalzl et al. 2019	14	63	2	14	21,9
Simpson et al. 1996	24	51	14	10	24
Strauß et al. 2006	23	52.9	5	12	29
	651	48.38	343	304	52.87
			53%	47%

**Table 2:** Most common trauma causes leading to Monteggia injuries

First author	Number of patients	High-energy fall	Low-energy fall	Traffic accident	Not specified fall	Work related	Other reasons
Egol et al. 2005	20			3	17		
Jungbluth et al. 2017	46	13	14	19			
Jupiter et al. 1991	13	3	10	0		0	
Klug et al. 2019	78	20	44	14		0	
Konrad et al. 2007	63			32	19	12	
Korner et al. 2004	49			28	16	5	
Laun et al. 2015	10	3	3	4			
Perez et al. 2002	54	0	21	32			1
Ring et al. 1998	48	8	29	5			6
Simpson et al. 1996	24	6	12	5			1
Strauß et al. 2006	6	4		2			
	411	57.00	133.00	144.00	52.00	17.00	8.00
		14%	32%	35%	13%	4%	2%

**Fig. 2:**
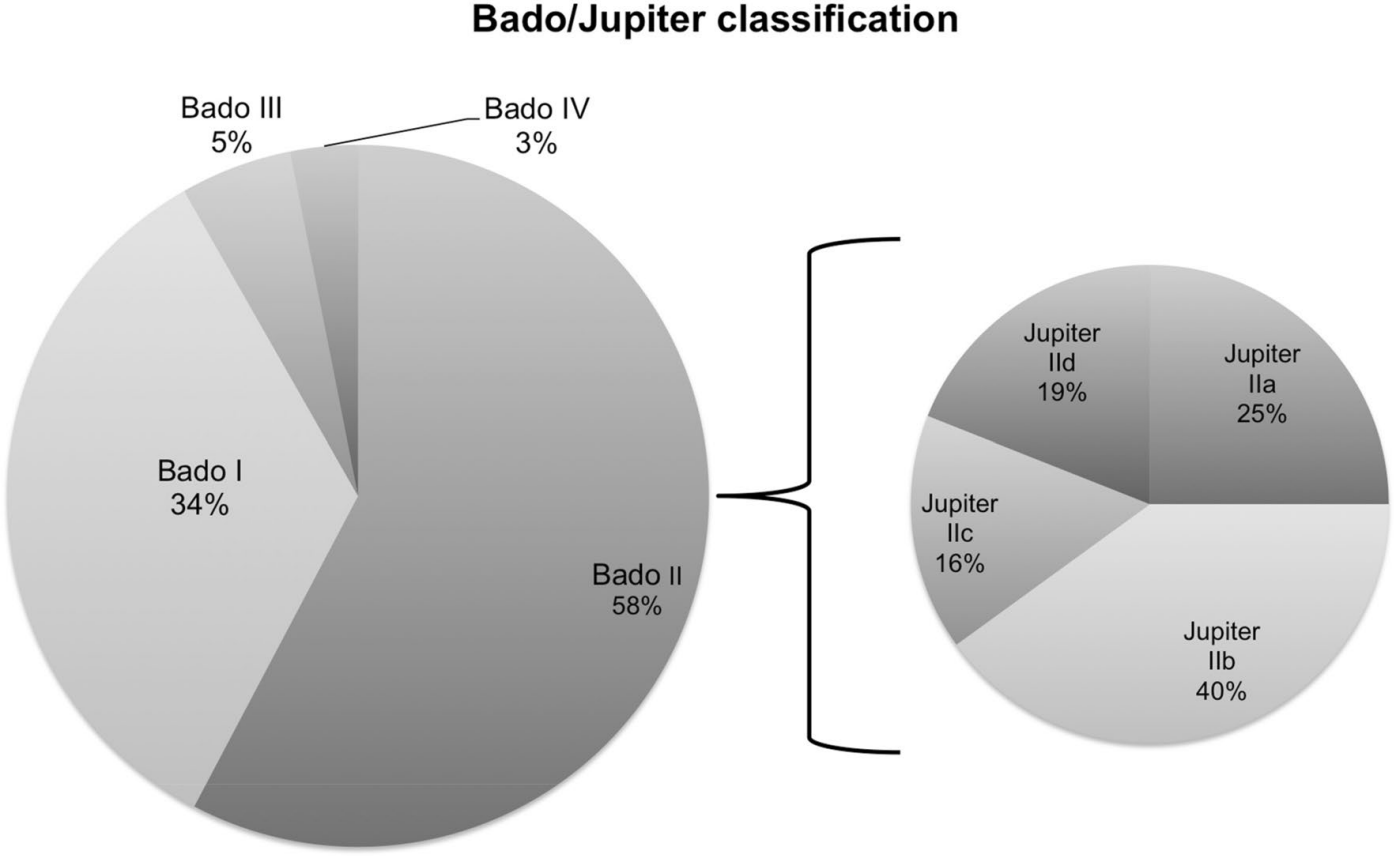
Bado classification of 651 patients and subclassification of 370 Bado type II fractures according to Jupiter

All patients were classified using the Bado classification for Monteggia fractures [4]. 31.5% were Bado type I fractures, 60.4% were Bado type II fractures. Bado type III and IV fractures are rare and represented only 5 and 3% of all Monteggia injuries, respectively. Thirteen of the 17 studies also provided information about the subclassification of Bado II fractures according to Jupiter [5]. There were 79 type IIa, 132 type IIb, 47 type IIc and 61 type IId injuries (Fig. 2).

290 radial head fractures were reported in 14 of the 17 publications. According to the Mason classification, 28% were classified as Mason type II fractures, 55% were classified as Mason type III fractures. Mason type I and IV constituted only 11 and 3% of all reported radial head fractures, respectively [6].

Seven publications additionally classified coronoid fractures. Jungbluth et al., Klug et al., Korner et al., Laun et al., Jung et al., and Strauss et al. used the Regan and Morrey classification [7–13]. Schmalzl and colleagues applied the O’Driscoll classification of coronoid process fractures [14, 15]. Altogether, there were 162 coronoid fractures reported.

There were 148 reported revisions, leading to a mean revision rate of 23% (0 to 63%). Main complications leading to additional surgery were ulna non-union (28%), limited range-of-motion, including elbow stiffness (22%), failed osteo-synthesis of ulna or radial head (13% and 11%), persistent instability (12%) and infection (9%). In 5% of the cases, revision was necessary due to other reasons that were not declared further (Table 3).

**Table 3:** Causes for revision surgery in Monteggia injuries

	Number of patients	Number of revisions	Revision rate	Ulna non-union	Infection	Failed osteo-synthesis ulna	Failed osteo-synthesis radial head	Limited ROM	Instability	Other reasons
Calderazzi et al. 2020	12	0	0	0	0	0	0	0	0	0
Eden et al. 2019	40	6	0.15	1	0	2	2	0	0	1
Egol et al. 2005	20	11	0.55	6	0	0	1	0	3	1
Guiton et al. 2009	11	2	0.18	2	0	0	0	0	0	0
Hamaker et al. 2017	119	23	0.19	4	5	0	0	3	1	1
Jung et al. 2020	27	17	0.63	0	0	0	5	5	0	0
Jungbluth et al. 2017	46	6	0.13	4	2	0	0	0	0	0
Jupiter et al. 1991	13	2	0.15	0	0	2	0	0	0	0
Klug et al. 2019	78	23	0.29	5	3	0	2	7	4	2
Konrad et al. 2007	63	10	0.16	6	0	0	2	2	0	0
Korner et al. 2004	49	14	0.29	4	1	0	1	5	3	0
Laun et al. 2015	10	0	0	0	0	0	0	0	0	0
Perez et al. 2002	54	14	0.26	4	1	0	0	4	4	0
Ring et al. 1998	48	12	0.25	1	0	6	3	1	1	0
Schmalzl et al. 2019	14	3	0.21	0	0	1	1	0	0	1
Simpson et al. 1996	24	5	0.21	0	0	3	0	2	0	0
Straus et al. 2006	6	2	0.33	2	0	0	0	0	0	0
Total	634	148.00	23%	37.00	12.00	14.00	17.00	29.00	16.00	6.00
percentage	105%	23%		28%	9%	11%	13%	22%	12%	5%

Ulna fractures were mainly treated using locking compression plates (LCP) or dynamic compression plates (including low contact dynamic compression plates, DCP or LCDCP), but ulna reconstruction plates, 1/3 tubular plates and tension wire were also used for internal fixation. In nine cases, external fixation was applied, and in one case, the surgeon decided to use a 6.5 mm cancellous bone screw (Table 4). Seven studies nearly exclusively used LCPs (222 LCPs in 227 cases), whereas nine studies mainly used LCDCPs or DCPs for Ulna reconstruction. Egol and colleagues utilized LCDCPs in 12 patients, LCDCP/DCPs in five patients and ulna reconstruction plates in three cases.

**Table 4:** Different treatment strategies and fixation devices for fixation of the Ulna in Monteggia injuries

	Number of patients	Ulna locking compression plate	Ulna LCDCP/DCP	Ulna reconstruction plate	1/3 tubular Plate	Ulna tension wire	6.5 mm cancellous bone screw	External fixation
Calderazzi et al. 2020	12	11	0	1	0	0	0	0
Eden et al. 2019	40	40	0	0	0	0	0	0
Egol et al. 2005	20	12	5	3	0	0	0	0
Guiton et al. 2009	11	0	10	0	0	0	0	1
Hamaker et al. 2017	119	0	119	0	0	0	0	0
Jung et al. 2020	27	24	0	0	0	3	0	0
Jungbluth et al. 2017	46	46	0	0	0	0	0	0
Jupiter et al. 1991	13	0	9	1	3	0	0	0
Klug et al. 2019	78	78	0	0	0	0	0	4
Konrad et al. 2007	63	0	52	0	0	11	0	0
Korner et al. 2004	49	0	15	21	3	8	0	2
Laun et al. 2015	10	10	0	0	0	0	0	0
Perez et al. 2002	54	0	35	11	3	3	1	1
Ring et al. 1998	48	0	37	2	0	3	0	0
Schmalzl et al. 2019	14	13	0	0	0	1	0	0
Simpson et al. 1996	24	0	24	0	0	0	0	0
Strauss et al. 2006	6	0	3	3	0	0	0	1
Total	634	234	309	42	9	29	1	9
Percentage	100%	37%	49%	7%	1%	5%	0%	1%

There was limited information about the approach used to treat the ulna fracture and, if present, the radial head fracture. Six studies used the posterior approach to reduce and fix the ulna fracture. To address radial head fractures, Schmalzl et al. and Simpson et al. used the Boyd approach, and Calderazzi et al., Hamaker et al. and Klug et al. used a lateral approach. Eleven studies did not provide any information regarding the approach they used.

In six studies, reconstruction of the lateral collateral ligament with suture anchors was performed if necessary [7, 8, 11, 12, 14, 16]. The other 11 studies did not provide any information whether they performed ligament reconstruction.

In 14 studies, a total of 255 radial head fractures were surgically treated. Open reduction and internal fixation were applied in 117 cases. Partial resection of radial head was performed in eight patients. Ninety-one radial head prostheses were implanted and 39 patients underwent total radial head resection (Table 5).

**Table 5:** Different treatment strategies and procedures addressing radial head fractures in Monteggia injuries

	ORIF	Partial resection	Prostheses	Resection
Calderazzi et al. 2020	2	2	5	1
Eden et al. 2019	19	0	1	1
Egol et al. 2005	3	0	4	0
Guiton et al. 2009	0	0	0	0
Hamaker et al. 2017	0	0	0	0
Jung et al. 2020	0	0	27	0
Jungbluth et al. 2017	15	0	22	0
Jupiter et al. 1991	3	0	1	6
Klug et al. 2019	26	0	19	7
Konrad et al. 2007	12	0	0	4
Korner et al. 2004	9	0	4	3
Laun et al. 2015	3	0	5	0
Perez et al. 2002	10	0	0	3
Ring et al. 1998	10	2	0	10
Schmalzl et al. 2019				
Simpson et al. 1996	2	4	0	4
Strauss et al. 2006	3	0	3	0
Total	117	8	91	39
Percentage	46%	3%	36%	15%

All but one publication reported about the range-of-motion (ROM) for extension-flexion and pronation-supination. Korner and colleagues reported the median ROM, and the other 15 studies reported mean range-of-motion. Disability of arm, shoulder and hand (DASH) score was used to asses clinical and functional outcome in 11 publications, Mayo elbow performance score (MEPS) was used in seven and Broberg and Morrey Score (BMS) in nine studies [17]. Mayo modified wrist score (MMWS) was reported in three publications. All publications report that revision surgery was associated with a worse outcome (Table 6).

**Table 6:** Overview of ROM and different outcome scores after Monteggia injuries

	Number of patients	ROM ex-flex	ROM pro-sup	MEPS	DASH	Broberg&Morrey	Mayo modified wrist score (MMWS)
Calderazzi et al. 2020	12	106.9	131.7	84.9	18.8		
Eden et al. 2019	40	117	136	84.0	28		
Egol et al. 2005	20	95	105		64.1	79.1	
Guiton et al. 2009	11	120.0	136.4	94.5	7.6	94.2	
Hamaker et al. 2017	119	121.36	138.40				
Jung et al. 2022	27	100.00	131.00	77.0	30.00		
Jungbluth et al. 2017	46	125.2	168.5	90.7	15.1	86.6	88.4
Jupiter et al. 1991	13	115.0	128.5			82.3	
Klug et al. 2019	78	114	155	88.9	14.7		88.1
Konrad et al. 2007	47	109.7	134.212766		17.4	87.2	
Korner et al. 2004	49	115 (Median)	130 (Median)				
Laun et al. 2015	10	121	160	89.2	20.1	86.5	86.5
Perez et al. 2002	54						
Ring et al. 1998	48	112	128			86	
Schmalzl et al. 2019	14	116	138	82.0	23.6	79	
Simpson et al. 1996	24	109.3	125.4				
Strauß et al. 2006	23	116.0	122.0		26.0	80.0	
Mean		114.90	140.24	87.99	18.02	85.50	88,08

**Fig. 3:**
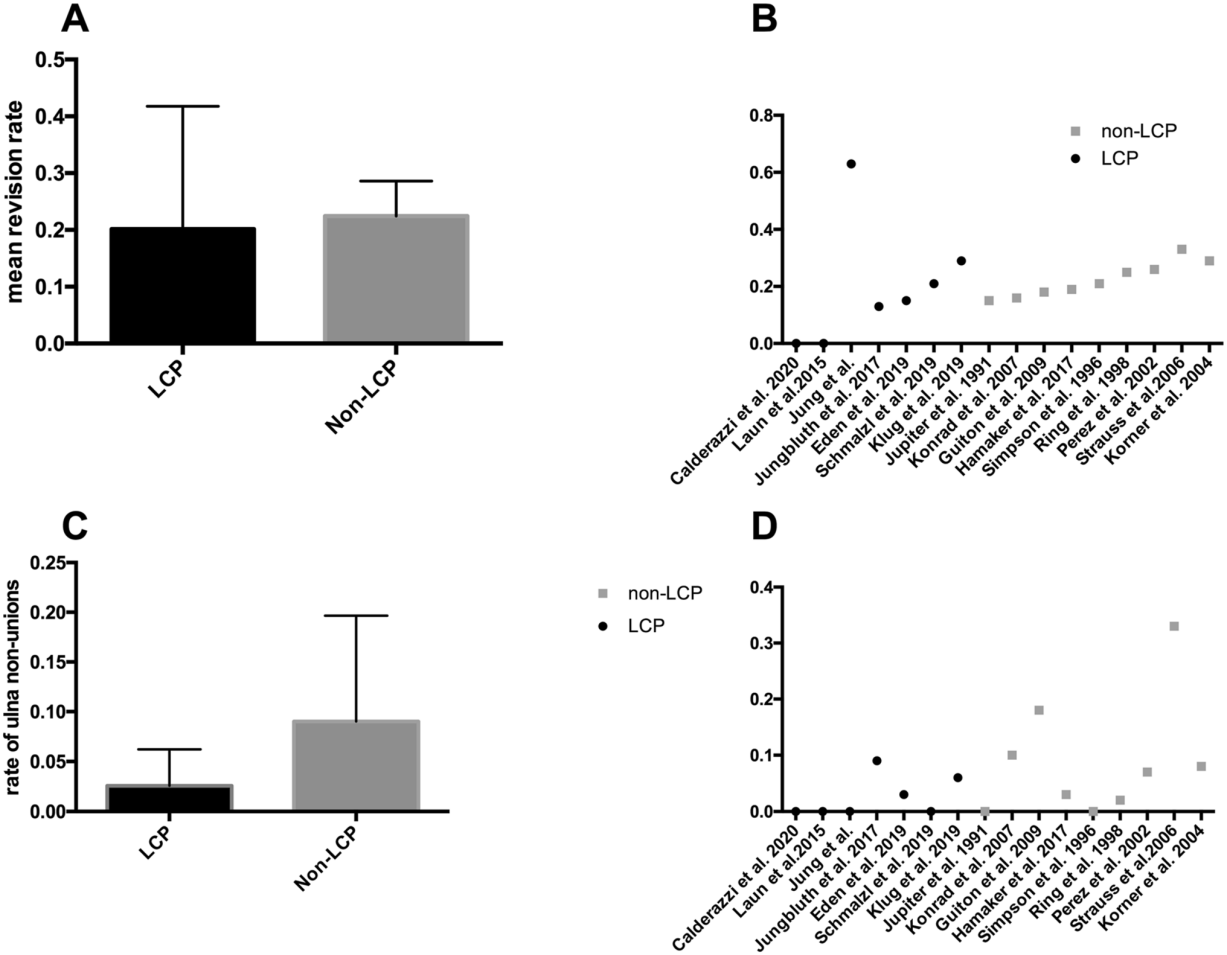
**A** Comparison of mean revision rates between publications using locking compression plates (LCP) for ulna fixation (*n* = 7) with publications not using locking compression plates (non-LCP; *n* = 9) rates. **B** overview of revision rates in 17 publications treating Monteggia injuries. **C** Comparison of ulna non-union rates between publications using locking compression plates (LCP) for ulna fixation (*n* = 7) with publications not using locking compression plates (non-LCP; *n* = 9). **D** Overview of ulna non-union rates in 17 publications treating Monteggia injuries

We hypothesized that surgical management of the ulna is crucial for preventing ulnar non-union and revision surgery in general. We compared seven studies mainly using locking compression plates (LCP in 222 of 227 cases) with nine studies mainly using non-locking fixation techniques. Mean revision rate of the LCP group was 0.2 (STD ± 0.08172; *n* = 7) compared to 0.22 (STD ± 002056; *n* = 9) in the non-LCP group. Mean rate of ulna non-union was 0.0257 (STD ± 0.01378; *n* = 7) in the LCP group and 0.09 (STD ± 0.0355; *n* = 9) in the non-LCP group. No significant difference with *p* < 0.05 was found using unpaired t test with Welch’s correction (Fig. 3).

## Discussion

In this systematic review on Monteggia injuries, we aimed to clarify the incidence of different injury patterns within Monteggia injuries, investigate the main reasons leading to revision surgery and explore which surgical treatments should be favored to achieve satisfactory clinical results. Ulna non-union (28%) and limited range-of-motion (22%) are the main reasons for revision surgery. The use of locking compression plates for ulna fixation might help reducing those complications.

The term “Monteggia fracture” or “Monteggia-like-lesion” does not clearly define injuries which makes it difficult to compare different publications regarding these subjects. Giannicola and colleagues [18] proposed a different approach to classify and determine Monteggia injuries, but the Bado/Jupiter classification remains the most common classification systems. The epidemiology within these classifications was not well investigated. Bado based his classification on a cohort of 22 adults and 18 children leading to a statistical distribution of 25% type I, 70% injuries [4]. Evans reported a distribution of 15% type I, 80% [19]. In our meta-analysis, Bado type II injuries remain the most common type (60.5%), but with a less frequent occurrence than previously reported. According to Jupiter et al., type IIc and IId injuries only account for 8% of the Bado type II injuries. Following our research, subtype IIb and IIa are the most common subtypes, but subtype IIc and IId seem to be more common than previously expected. Our meta-analysis provides more precise data about the epidemiology and incidents of different subtypes of Monteggia injuries than previous studies.

The mean revision rate in our meta-analysis was 23%, indicating that Monteggia injuries are still difficult to treat. By analyzing reasons for revision, we could point out that ulna non-union (28%) and failed osteo-synthesis of ulna (11%) were common reasons for revision surgery. These findings suggest a lack of stability of the ulna fixation and therefore raise the question for the best treatment strategy for theses fractures. Biomechanical investigations show that fixation locking compression plates show less axial displacement than reconstruction plates and 1/3 tubular plates and therefore suggest that locking compression plates are favorable in complex ulna fractures [20]. Eden and colleagues used different types of locking compression plates including variable-angle locking double mini-plates, straight and pre-contoured plates with overall good results [21]. Comparing the LCP with the non-LCP group in our meta-analysis, we could see a lower revision rate (20% vs. 22%) and a lower rate of ulna non-unions in the LCP group (2.6% vs. 9%). Nevertheless, no significant differences could be found, presumably due to the underpowered t test. Considering the current biomechanical investigations and the favorable tendencies in our meta-analysis, we recommend using locking compression plates for the fixation of Monteggia injuries.

Complications in radial head osteo-synthesis (13%) and persistent instability (12%) are also common reasons for revision surgery in Monteggia injuries. Since postero-lateral instability of the radial head is one of the main reasons for persistent instability, the management of radial head fractures seems crucial to avoid instability and failed osteo-synthesis [9, 21, 22]. Especially, Mason III fractures raise the question whether they should be treated via ORIF, prostheses or radial head resection. Klug et al. showed that reconstruction of Mason III fractures in Monteggia injuries leads to better clinical results than resection or arthroplasty. There was no significant difference between radial head resection and radial head arthroplasty. Thus, more research is needed to clarify whether radial head arthroplasty or radial head resection leads to significant differences for the overall outcome after Monteggia injuries.

## Conclusion

In conclusion, we could present new insights regarding the epidemiology of Monteggia injuries regarding the Bado/Jupiter classification, could show that revisions in Monteggia injuries are still frequent and highlight the main complications leading to revision surgery. There are hints that using locking compression plates for ulna fixation could lead to less revision surgery and prevent ulna non-union, but more research is necessary to support these tendencies. Coronoid fractures and reconstructable radial head fractures should be addressed with open reduction and internal fixation. Whether radial head arthroplasty shows significant advantages over radial head resection remains debatable.

## References

[1] Rehim SA, Maynard MA, Sebastin SJ, Chung KC (2014). Monteggia fracture dislocations: a historical review. J Hand Surg.

[2] Josten C, Freitag S (2009). Monteggia and monteggia-like-lesions: classification, indication, and techniques in operative treatment. Eur J Trauma Emerg Surg.

[3] Giannicola G, Sacchetti FM, Greco A, Cinotti G, Postacchini F (2010). Management of complex elbow instability. Musculoskelet Surg.

[4] Bado JL (1967). The Monteggia lesion. Clin Orthop.

[5] Jupiter JB, Leibovic SJ, Ribbans W, Wilk RM (1991). The posterior Monteggia lesion. J Orthop Trauma.

[6] Mason ML (1954). Some observations on fractures of the head of the radius with a review of one hundred cases. Br J Surg.

[7] Jungbluth P (2018). The challenge of Monteggia-like lesions of the elbow: mid-term results of 46 cases. Bone Jt J.

[8] Klug A, Konrad F, Gramlich Y, Hoffmann R, Schmidt-Horlohé K (2019). Surgical treatment of the radial head is critical to the outcome of Monteggia-like lesions. Bone Jt J.

[9] Korner J (2004). Monteggia injuries in adults: critical analysis of injury pattern, management, and results. Unfallchirurg.

[10] Laun R, Wild M, Brosius L, Hakimi M (2015). Monteggia-like lesions—treatment strategies and one-year results. GMS Interdiscip Plast Reconstr Surg DGPW.

[11] Jung M, Groetzner-Schmidt C, Porschke F, Grützner PA, Guehring T, Schnetzke M (2020). Monteggia-like lesions in adults treated with radial head arthroplasty-mid-term follow-up of 27 cases. J Orthop Surg.

[12] Strauss EJ, Tejwani NC, Preston CF, Egol KA (2006). The posterior Monteggia lesion with associated ulnohumeral instability. J Bone Joint Surg Br.

[13] Regan W, Morrey B (1989). Fractures of the coronoid process of the ulna. J Bone Joint Surg Am.

[14] Schmalzl J, Sadler N, Feucht M, Gerhardt C, Lehmann LJ (2019). Monteggia-like lesions. Obere Extrem.

[15] O’Driscoll SW, Jupiter JB, Cohen MS, Ring D, McKee MD (2003). Difficult elbow fractures: pearls and pitfalls. Instr Course Lect.

[16] Calderazzi F (2020). Monteggia-like lesions: preliminary reports and mid-term results of a single center. Acta Bio-Medica Atenei Parm.

[17] Hudak PL, Amadio PC, Bombardier C (1996). Development of an upper extremity outcome measure: the DASH (disabilities of the arm, shoulder and hand) [corrected]. The Upper Extremity Collaborative Group (UECG). Am J Ind Med.

[18] Giannicola G, Scacchi M, Sacchetti FM, Cinotti G (2013). Clinical usefulness of proximal ulnar and radial fracture-dislocation comprehensive classification system (PURCCS): prospective study of 39 cases. J Shoulder Elbow Surg.

[19] Evans EM (1949). Pronation injuries of the forearm, with special reference to the anterior Monteggia fracture. J Bone Joint Surg Br.

[20] Wegmann K (2016). Reconstruction of Monteggia-like proximal ulna fractures using different fixation devices: a biomechanical study. Injury.

[21] Eden L, Frey SP, Gilbert F, Jordan MC, Fenwick A, Meffert RH (2020). Anatomically shaped locking plates for radial head and olecranon fracture fixation in Monteggia-like lesions. Technol Health Care.

[22] Llusà Perez M, Lamas C, Martínez I, Pidemunt G, Mir X (2002). Monteggia fractures in adults. Review of 54 cases. Chir Main.

[23] Egol KA, Tejwani NC, Bazzi J, Susarla A, Koval KJ (2005). Does a monteggia variant lesion result in a poor functional outcome?: a retrospective study. Clin Orthop.

